# Brief Report: Switching to Long-Acting CAB/RPV: Data From an Italian Monocentric Cohort

**DOI:** 10.1097/QAI.0000000000003501

**Published:** 2024-10-23

**Authors:** Maddalena Matone, Marco Piscaglia, Andrea Giacomelli, Davide Moschese, Amedeo Capetti, Giacomo Pozza, Lucia Galli, Spinello Antinori, Andrea Gori, Giuliano Rizzardini, Maria Vittoria Cossu

**Affiliations:** a Department of Infectious Diseases, Unit I, ASST Fatebenefratelli Sacco, Luigi Sacco Hospital, Milan, Italy;; b Centre for Multidisciplinary Research in Health Science (MACH), University of Milan, Milan, Italy;; c III Division of Infectious Diseases, ASST Fatebenefratelli Sacco, Luigi Sacco Hospital, Milan, Italy;; d Department of Biomedical and Clinical Sciences, Università Degli Studi di Milano, Milan, Italy; and; e Department of Infectious Diseases, Unit II, ASST Fatebenefratelli Sacco, Luigi Sacco Hospital, Milan, Italy.

**Keywords:** adherence, long-acting antiretroviral therapy, cabotegravir, discontinuation, HIV, rilpivirine

## Abstract

**Background:**

Cabotegravir (CAB)/rilpivirine (RPV) is the first long-acting injectable (LAI) antiretroviral therapy approved for virologically suppressed adults with HIV-1.

**Setting:**

Italian single centre cohort.

**Methods:**

We conducted a retrospective observational study to assess the durability, adherence to the prescribed injection schedule, and reasons for discontinuation of CAB/RPV LAI administered every 8 weeks (Q8W).

**Results:**

One hundred thirty-eight patients were included with a median observation period of 43 weeks [interquartile range (IQR) 34–47 weeks]. Of these, 32 (23.2%) were female, and the median age was 51 years (IQR 40–58 years). Twelve patients (8.7%) discontinued CAB/RPV LAI treatment with a median time to discontinuation of 21 weeks (IQR 12–35 weeks), and 92.8% of the injections occurred within the CAB/RPV LAI schedule. The most common reason for discontinuation was injection-related pain (5/12). No confirmed virological failure occurred during the period of observation with 3 individuals who experienced virological blips.

**Conclusions:**

Our findings showed that CAB/RPV LAI Q8W is tolerated well in clinical practice, with high adherence to the injection schedule and few discontinuations mainly related to injection site–related pain.

## INTRODUCTION

The continuous evolution of antiretroviral therapies (ARTs) has significantly changed the landscape of HIV treatment, offering improved efficacy, tolerability, and adherence. However, despite these advancements, long-term adherence to daily oral therapy remains challenging for people with HIV (PWH).^1^ Recent years have seen a significant breakthrough with the introduction of long-acting injectable (LAI) regimens, offering an alternative to the conventional daily oral treatment approach.^2^ Cabotegravir (CAB)/rilpivirine (RPV) is the first LAI ART approved for virologically suppressed PWH-1.^3^ This innovative approach marks a crucial step forward in addressing adherence issues and enhancing the overall efficacy of HIV treatment strategies.

Clinical trials, including LATTE-2,^4^ Antiretroviral Therapy as Long-Acting Suppression, and First Long-Acting Injectable Regimen,^5^ have substantiated the efficacy and safety of CAB/RPV LAI administered every 4 weeks (Q4W). Furthermore, findings from the Antiretroviral Therapy as Long-Acting Suppression-2M trial demonstrate that administering CAB/RPV LAI every 8 weeks (Q8W) maintains noninferiority compared with the Q4W schedule.^6^

Although clinical trials provide essential information regarding the safety and efficacy of medical interventions, real-life scenarios introduce a variety of factors that can significantly impact patient adherence and, consequently, treatment outcomes.

Observational studies in real-world settings have corroborated the pharmacokinetic profiles and virological outcomes observed in clinical trials.^7,8^ Moreover, adherence to CAB/RPV LAI, both Q4W and Q8W, has shown promising results in these studies, with low rates of injection delays and regimen discontinuations noted.^8,9^ Despite these positive findings, real-life studies specifically focusing on the Q8W schedule are underrepresented. In addition, these studies have observed that adverse events associated with CAB/RPV LAI are generally contained, with the most common being injection site–related pain, which rarely leads to discontinuation of the regimen.^9,10^

Thus, there remains a need for further investigation, particularly focusing on the Q8W dosing schedule in routine clinical practice. In this context, our study aims to assess the durability, adherence to the prescribed injection schedule, and reasons for discontinuation of CAB/RPV LAI Q8W in a single-centre cohort of PWH in Italy.

## METHODS

### Study Design and Setting

This is a retrospective observational study conducted at a third-level centre (Department of Infectious Diseases, ASST Fatebenefratelli Sacco, in Milan, Italy).

We included consecutive PWH who (1) switched to CAB/RPV LAI, (2) started the LAI regimen between November 1, 2022, and April 30, 2023, and (3) had a viral load (VL) of <50 cp/mL for at least 6 months before the switch. We followed the included patients until December 31, 2023 (end of study period). The switch to CAB/RPV LAI was proactive and patient driven at our center in the absence of a documented previous regimen-related toxicity. During the initial 3 months of the study, 2 physicians assessed eligibility by reviewing patient records. Subsequently, clinicians referred eligible patients directly to CAB/RPV LAI. Eligibility was determined based on comprehensive medical history review, assessment of genotype and virological failure, evaluation of previous ART exposure, consideration of potential drug interactions, assessment of hepatitis B serostatus, confirmed undetectable VL at baseline, and no prior virological failure to CAB or RPV. For the purposes of this study, baseline (BL) is defined as the date of the first intramuscular injection of CAB/RPV, following any oral lead-in phase if applicable.

Following the commercial availability of this new treatment option, our center implemented a novel approach by establishing a nursing clinic adjacent to the medical clinics. Patients are referred by their HIV physician to the nursing clinic for injections, allowing for concurrent blood samples and follow-up visits. This approach aims to reduce hospital visits to 6 per year for clinically and virologically stable patients. Injections are scheduled in advance for a six-month period, and the nursing clinic staff provides the patient list.

All PWH with stable virological control who switched to CAB/RPV LAI followed an 8-week schedule. Eligible patients were given the opportunity to choose to switch to injectable treatment. Virological failure was defined as VL >50 cp/mL in 2 consecutive measurements taken 4 weeks apart or VL > 1000 cp/mL in a single measurement, whereas virological blip was defined as VL >50 and <1000 cp/mL in a single measurement followed by measurements less than 50 cp/mL without any change in treatment.^11^

### Data Collection

Data were collected through manual review of clinical records. Baseline data were comprehensively collected, encompassing demographic and physiological characteristics, medical history, and details of any concomitant medical conditions or therapies, including previous ART regimens and oral lead in. VL was determined at 4 weeks after the start of therapy and, because patients already had a VL of < 50 cp/mL at baseline and at 24 and 48 weeks after the first injection. If VL > 50 cp/mL was detected, a follow-up VL test was performed 4 weeks later to confirm virological status and to guide further clinical decisions. All VL measurements taken during the study period were included in the analysis. The appropriate time window for CAB/RPV LAI injections was defined as 28 ± 7 days from the previous injection for the second injection and as 56 ± 7 days for the further administrations. For patients who discontinued treatment, the date of the last effective injection was considered the date of discontinuation. Reasons for discontinuation were collected by systematic analysis of medical records.

### Outcomes of Interest

The primary outcome was the discontinuation of CAB/RPV LAI for any reason. The secondary outcome was adherence to the injection schedule.

### Statistical Analysis

Continuous variables were described using appropriate summary statistics, such as mean or median [with SD or interquartile range (IQR), as appropriate], whereas categorical variables were expressed as frequencies and percentages. All causes discontinuation was estimated by means of Kaplan–Meier curves.

Study data were collected and managed using REDCap electronic data capture tools hosted at ASST-FBF-SACCO. Statistical analyses were performed with IBM SPSS Statistics, v. 28.0.

### Ethical Statement

The study protocol received approval from the Ethical Committee of Luigi Sacco Hospital (Protocol 0011903/2023–March 16, 2023), and no external funding was received for this research endeavor.

## RESULTS

We included 138 patients who received CAB/RPV LAI with a median time of observation of 43 weeks (IQR 34–47 weeks). Of these, 32 were female (23.2%), the median age was 51 years (IQR 40–58 years), and 64 (47%) were taking almost one comedication.

Out of 138 individuals, 88 (63.8%) had ever been exposed to INSTIs (integrase strand transfer inhibitors), 81 (58.7%) to NNRTIs (nonnucleoside reverse transcriptase inhibitors), and 60 (43.5%) to PIs (protease inhibitors). Before switching to CAB/RPV LAI, 107 patients (77.5%) were on an INSTI-containing regimen, 44 (31.9%) from a NNRTI-containing regimen, and 5 (6.3%) from a PI-containing regimen. Median time of VL <50 cp/mL before the switch was 10 years (IQR: 6–16 years).

**FIGURE 1.**
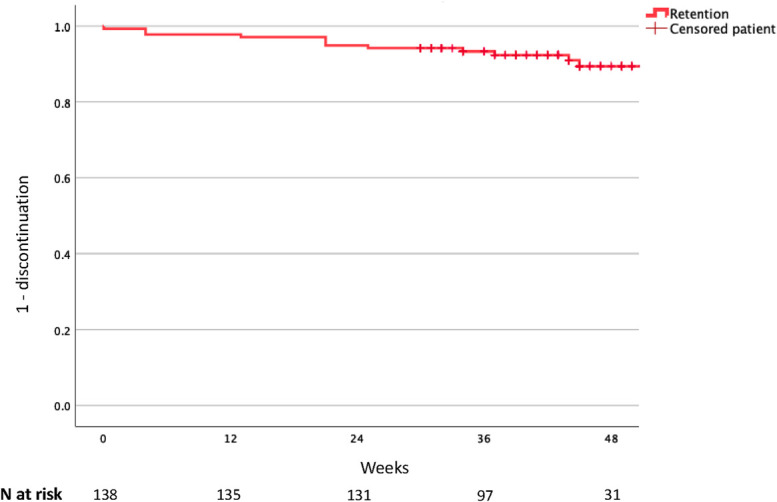
The Kaplan–Meier curve depicts the probability of treatment continuation over time up to 48 weeks. Vertical dashes indicate censored data (end of observation while on therapy).

Other clinical characteristics of the cohort are resumed in Table 1. In our study, 12 PWH (8.7%) discontinued the treatment (Fig. 1), with a higher proportion of female PWH compared with male PWH (7/32, 21.7% versus 5/106, 4.7%; risk ratio 5.7; *P*= 0.03). The median time to discontinuation was of 21 weeks (IQR 12–35 weeks), and the median number of injections before discontinuation was 4 (IQR 3–5). The probability of adherence after 48 weeks of LAI was estimated at 89.4% (95% confidence interval 83.3% to 95.5%). Reasons for discontinuation among these 12 individuals included injection-related pain (n = 5), evidence of hepatitis B virus infection retrospectively identified postinitiation of CAB/RPV LAI (n = 1), clinical decision after an episode of major depression (n = 1), pregnancy (n = 1), implant of silicon prosthesis (n = 1), drug–drug interactions (n = 1), evidence of RPV resistance–associated mutations after reexam of genotypic resistance test (n = 1), and logistic reasons (n = 1). We recorded 3 (2.2%) virological blips (all< 200 cp/mL) and no virological failures during the study period.

**TABLE 1.** Description of the Population

	N = 138
Demographics	
Female (%)	32 (23.2)
Median age (IQR)	51 (40–58)
MSM (%)	80 (58)
IVDU (%)	7 (5.1)
No. of comedications (%)	
0	72 (52.9)
1	23 (16.9)
2 or more	41 (30.1)
BMI > 30 kg/m^2^	12 (8.7)
BMI > 35 kg/m^2^ and < 40 kg/m^2^	1 (0.72)
BMI > 40 kg/m^2^	0 (0)
Median time from diagnosis (IQR)	13.0 (8–20)
AIDS (%)	17 (12.8)
No. of previous ARTs (IQR)	4 (3–5)
NNRTI exposure (%)	81 (58.7)
INSTI exposure (%)	88 (63.8)
PI exposure (%)	60 (43.5)
No oral lead in (%)	23 (18.3)
BL CD4 (IQR)	755 (597–895)
BL CD4 < 500 cells/mm^3^ (%)	24 (17.4)
Results	
Median N of injections (IQR)	6 (5–7)
Discontinuation (%)	12 (8.7)
Injection in adequate window (%)	629 (96.3)
Individual N of delays (%)	
0	128 (92.8)
1	9 (6.5)
2 or 3	1 (0.7)

Continuous variables are described using appropriate summary statistics, such as mean or median (with SD or IQR, as appropriate), whereas categorical variables are expressed as frequencies and percentages.

BMI, body mass index; BL, blood lymphocytes; INSTI, integrase strand transfer inhibitors; IVDU, intravenous drug users; MSM, men having sex with men; NNRTI, nonnucleoside reverse transcriptase inhibitors; PI, protease inhibitors.

**FIGURE 2.**
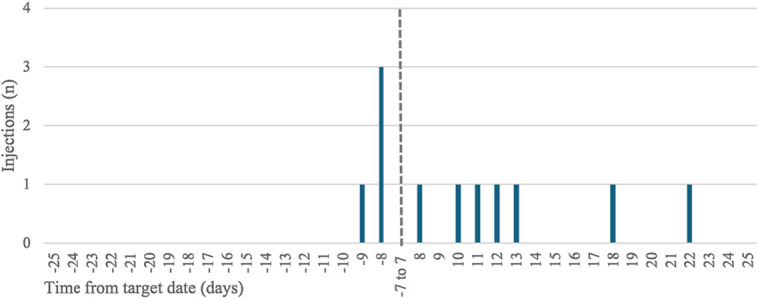
Distribution of injection delays beyond the ±7-day window.

Analysis of adequacy of time window of injections showed that 92.8% (128/138) of patients did not experience delayed injections. Among the total injections, 629 (96.3%) were administered within the adequate ±7-day window. Nine patients had one injection delay, whereas one patient experienced 2 delays. Most injections were administered within a few days of the target window, with only 2 patients experiencing significant delays greater than +13 days, including one instance of a +22-day delay (Fig. 2).

Finally, a patient developed significant gluteal pain after the second injection of CAB/RPV LAI. To manage this pain, the patient was placed on oral bridging for 21 days, postponing the third injections. After this period, injections were resumed according to the original schedule, with the first 2 doses given 28 days apart. The patient did not experience a virological blip.

## DISCUSSION

CAB/RPV became available at our centre in November 2022. Over the following 6 months, 138 patients switched to CAB/RPV LAI Q8W. The discontinuation rate, particularly less than 10% within our monocentric cohort, underscores the overall positive outcome of the implementation strategy at our centre. A significant contributing factor to discontinuation, identified in half of the cases, was the occurrence of injection site pain.

Furthermore, most injections were administered within the correct time window, demonstrating the feasibility and patient compliance with the proposed treatment schedule in the setting of our hospital. This adherence rate is consistent with other real-life studies, which generally report adherence rates above 90% for the CAB/RPV LAI regimen.^8,9^ This alignment with broader findings underscores the reliability and practicality of the proposed injection schedule.^1,2,12^ Notably, the establishment of our nursing clinic adjacent to the medical clinics played a pivotal role in ensuring timely administration of injections, thereby enhancing patient compliance and feasibility from a management perspective. By assuming responsibility for scheduling and overseeing injections, the nursing clinic effectively alleviated the workload of the medical team, allowing them to focus on other clinical responsibilities.

The absence of virological failure during the study period is an important finding in the confirmation of the efficacy of CAB/RPV LAI in the maintenance of viral suppression. This is consistent with findings from clinical trials^4–6^ but reinforces its real-world applicability.^7–10^

Interestingly, we observed a higher discontinuation rate in female patients in our cohort. However, given the small sample size, this may be a chance finding. Another recent larger Italian study did not report a similar observation regarding discontinuation rates among women, whereas our study included a higher proportion of female patients.^13^

However, our study has several limitations that warrant consideration. Being retrospective, it reflects real-life clinical scenarios where missing data, such as incomplete VL measurements for some patients, may affect the robustness of findings. Dependence on existing medical records introduces potential biases in outcome interpretation and understanding of patient management nuances. In addition, changes in patient selection criteria over time by treating physicians may have led to oversights in clinical aspects like the identification of hepatitis B virus infection or preexisting resistance-associated mutations. Furthermore, the relatively small sample size limits the generalizability of our findings and may contribute to observed higher discontinuation rates among female patients. The median observation period of 43 weeks might not capture the long-term effects of CAB/RPV LAI adequately, given its intended use as a prolonged therapy. Moreover, focusing exclusively on CAB/RPV LAI limits our ability to compare its performance with other ARTs, potentially overlooking alternative treatment considerations.

Future research with a longer observation period, multicentre design and broader scope may provide a more comprehensive understanding of the performance of CAB/RPV LAI.

## CONCLUSIONS

In conclusion, our findings showed that CAB/RPV LAI Q8W is well-tolerated in clinical practice, with high adherence to the injection schedule and few discontinuations mainly related to injection site–related pain. Further studies about practical strategies to minimize injection-related pain are needed.
